# P14 A case of worsening scleroderma-associated interstitial lung disease in pregnancy – treated with tocilizumab

**DOI:** 10.1093/rap/rkae117.045

**Published:** 2024-11-05

**Authors:** Roobala Kaliannan Periyasami, Suzanne Hamilton, Eiphyu Htut, Poonam Sharma

**Affiliations:** Department of Rheumatology, Northwest Anglia NHS Foundation Trust, Peterborough, United Kingdom; Department of Obstetrics and Gynaecology, Northwest Anglia NHS Foundation Trust, Peterborough, United Kingdom; Department of Rheumatology, Cambridge Universtiy Hospitals NHS Foundation Trust, Cambridge, United Kingdom; Department of Rheumatology, Northwest Anglia NHS Foundation Trust, Peterborough, United Kingdom

## Abstract

**Introduction:**

Systemic sclerosis is a rare, multi-system connective tissue disorder, predominantly affecting women of reproductive age, with remarkable heterogeneity in organ involvement. Pulmonary involvement, such as interstitial lung disease (ILD) and pulmonary hypertension, remains the leading cause of death. There have been reports of disease progression during pregnancy and trials are ongoing to establish a safe treatment regimen for patients with exacerbation of ILD during pregnancy. We present an interesting case where interleukin-6 (IL-6) inhibitor, tocilizumab, was successfully used to control inflammation in the lung during pregnancy, resulting in a positive outcome for both the mother and the baby.

**Case description:**

Our patient was diagnosed with diffuse cutaneous systemic sclerosis with overlapping facial dermatomyositis at the age of 25. She was found to have anti Scl-70 antibody, anti-Ro antibody and weakly positive anti Jo–1 antibody. On review, she was noted to have significant Raynaud’s disease, fixed flexion deformity at the proximal interphalangeal joints as a consequence of digital ulcers. At the age of 29, minor lung fibrosis was detected in the CT, with pulmonary function tests showing FEV1 at 87%, FVC at 87%, and TLCO at 73%. She was managed with mycophenolate mofetil, hydroxychloroquine, prednisolone, sildenafil, omeprazole, and vitamin D. During active disease phases with digital ulcerations, she received iloprost and bosentan.

She had her first child aged 30 without complications while off mycophenolate mofetil. Planning for another pregnancy at 34, she was advised to stop mycophenolate mofetil. During her 21st week of pregnancy, she presented with palpitations, non-productive cough, and shortness of breath. A limited sequence CTPA revealed diffuse ground-glass opacity with mid and lower zone predominance, indicating progressive inflammatory changes compared to the previous imaging. Repeat lung function tests showed significant deterioration: FEV1 at 75%, FVC at 68%, and TLCO at 57%. Her echocardiogram was normal with no evidence of pulmonary hypertension.

Despite the increase in her prednisolone to 10 mg and the initiation of azathioprine, she continued to experience dyspnoea at rest. After multidisciplinary discussions and review of guidelines and literature, the decision was made to use tocilizumab to stabilise her lung disease. She received two doses, one in the second and another in the third trimester. She safely delivered a healthy baby via planned C-section, and her symptoms significantly improved postpartum, allowing her to switch back to her usual mycophenolate mofetil.

**Discussion:**

The incidence of ILD is highest within the first five years of a systemic sclerosis diagnosis. Patients with anti-topoisomerase antibodies are at a higher risk of developing ILD. Synthetic DMARDs, most commonly mycophenolate mofetil, are used to induce remission, but they have teratogenic side effects in pregnancy. The FDA approved tocilizumab, a humanised monoclonal antibody against the IL–6 receptor, for management of ILD associated with systemic sclerosis.

Exacerbation of ILD during pregnancy is expected, with limited case reports on using non-teratogenic DMARDs and biological agents. For systemic sclerosis-ILD, the American College of Rheumatology conditionally recommends tocilizumab as a first-line treatment option. A meta-analysis suggests tocilizumab significantly prevents worsening of FVC, though it shows no significant improvement in skin disease control. The evidence regarding the teratogenicity of IL-6 inhibitors during pregnancy is limited. While it is advisable to discontinue IL-6 inhibitors at conception, exposure during pregnancy is unlikely to cause harm. If used in the third trimester, precautions such as avoiding live vaccines for the infant until six months of age are recommended. Maternal use of IL-6 inhibitors appears compatible with breastfeeding based on limited evidence.

Pre-conception counselling should optimise disease control before pregnancy, including advice on timing, drug therapy, and contraception. Women planning pregnancy should avoid incompatible drugs, and the risks and benefits of treatment should be discussed. Contraindicated immunomodulatory drugs should be switched to pregnancy-compatible alternatives before conception. In severe maternal disease, controlling the disease takes priority over potential foetal outcomes, and biologic DMARDs may be continued if necessary.

In this case, the patient experienced symptoms with significant deterioration in CT and lung function tests after stopping mycophenolate mofetil to conceive. After consulting with regional experts, tocilizumab was obtained through Trust funding, which helped to limit progression of disease.

**Key learning points:**

• Early treatment is cruical for improving prognosis. The goal of treatment strategies is to achieve remission by halting disease progression and ideally reversing some disease-related changes.

• There are studies supporting the efficacy of tocilizumab in improving lung function tests for patients with systemic sclerosis. In the above case, the off-label use of tocilizumab saved the life of this pregnant woman. This suggests that tocilizumab may help stabilise inflammatory lung disease during pregnancy, indicating its potential use in managing severe maternal disease if other pregnancy-compatible drugs are not suitable. The decision to use tocilizumab in pregnancy involves careful consideration of the risks and benefits, with input from a multidisciplinary team to ensure comprehensive patient care.

